# Nanosecond-Pulsed DBD Plasma-Generated Reactive Oxygen Species Trigger Immunogenic Cell Death in A549 Lung Carcinoma Cells through Intracellular Oxidative Stress

**DOI:** 10.3390/ijms18050966

**Published:** 2017-05-03

**Authors:** Abraham Lin, Billy Truong, Sohil Patel, Nagendra Kaushik, Eun Ha Choi, Gregory Fridman, Alexander Fridman, Vandana Miller

**Affiliations:** 1 C. & J. Nyheim Plasma Institute, Drexel University, Philadelphia, PA 19104, USA; agl46@glink.drexel.edu (A.L.); billytruong2016@gmail.com (B.T.); ssp82@drexel.edu (S.P.); gf33@drexel.edu (G.F.); af55@drexel.edu (A.F.); 2 Plasma Bioscience Research Center, Kwangwoon University, Seoul 139791, Korea; kaushik.nagendra@gmail.com (N.K.); ehchoi@kw.ac.kr (E.H.C.)

**Keywords:** non-thermal plasma, cancer immunotherapy, immunogenic cell death, oxidative stress, calreticulin, adenosine triphosphate, plasma immunotherapy, nanosecond-pulsed dielectric barrier discharge

## Abstract

A novel application for non-thermal plasma is the induction of immunogenic cancer cell death for cancer immunotherapy. Cells undergoing immunogenic death emit danger signals which facilitate anti-tumor immune responses. Although pathways leading to immunogenic cell death are not fully understood; oxidative stress is considered to be part of the underlying mechanism. Here; we studied the interaction between dielectric barrier discharge plasma and cancer cells for oxidative stress-mediated immunogenic cell death. We assessed changes to the intracellular oxidative environment after plasma treatment and correlated it to emission of two danger signals: surface-exposed calreticulin and secreted adenosine triphosphate. Plasma-generated reactive oxygen and charged species were recognized as the major effectors of immunogenic cell death. Chemical attenuators of intracellular reactive oxygen species successfully abrogated oxidative stress following plasma treatment and modulated the emission of surface-exposed calreticulin. Secreted danger signals from cells undergoing immunogenic death enhanced the anti-tumor activity of macrophages. This study demonstrated that plasma triggers immunogenic cell death through oxidative stress pathways and highlights its potential development for cancer immunotherapy.

## 1. Introduction

Methods to induce immunogenic cell death (ICD), where dying cells emit immuno-stimulatory signals, are being actively explored for cancer immunotherapy [1,2,3,4,5]. Cancerous cells undergoing ICD recruit and stimulate antigen presenting cells (APCs), key immune cells required for the initiation of an adaptive immune response [4,5,6]. This leads to the subsequent development and activation of tumor-specific effector T cells and memory T cells [7,8,9]. Thus, cancer is suppressed systemically and long-term protection against cancer recurrence is possible [8,9].

While mechanisms that promote ICD are under investigation, molecules characteristic of ICD known as damage-associated molecular patterns (DAMPs), have been identified [10,11]. These molecules are exposed on the cell surface or are secreted by cells undergoing autophagy, endoplasmic reticulum (ER) stress, or oxidative stress from reactive oxygen species (ROS) [6,10,11,12,13]. DAMPs that are characteristically associated with ICD are: surface-exposed calreticulin (ecto-CRT), secreted adenosine triphosphate (ATP), released high mobility group protein B1 (HMGB1), and surface-exposed heat shock protein 90 (HSP90) and HSP70 [13,14,15,16]. Of these, CRT and ATP are identified as the major predictors of the immuno-stimulatory capacity of anti-cancer therapeutics [15,16,17,18]. Retrospective evaluation of experimental and clinical data has led to the identification of several ICD inducers, including some chemotherapeutics (anthracyclines and oxaliplatin), ionizing radiation, and photodynamic therapy with hypericin [1,2,3]. At the same time, new ICD inducers, such as non-thermal plasma, are also being investigated.

**Figure 1:**
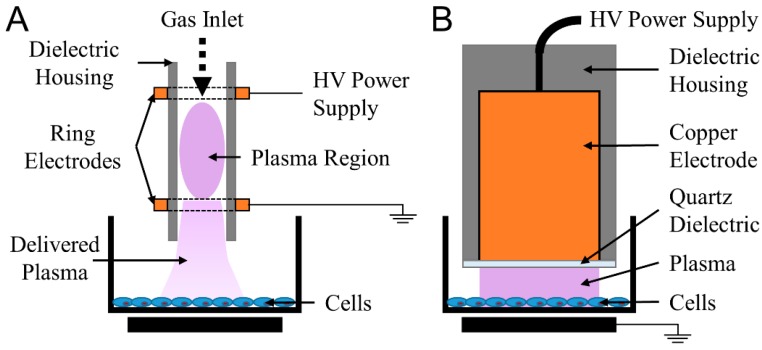
Two major families of non-thermal, atmospheric pressure plasma devices for biomedical applications are jets and dielectric barrier discharges (DBDs) (**A**) Jets remotely generate bulk plasma and plasma species are often transported to the target via a carrier gas; (**B**) DBDs plasma use the target as a grounding electrode to generate plasma directly onto cells and tissue.

Plasma, known as the fourth state of matter, is an ionized gas composed of electrons, various ions, electronically excited atoms and neutral atoms and molecules [19]. Two major families of devices for generating non-thermal plasma at atmospheric pressure for biological and medical applications are (Figure 1): (1) plasma jets and (2) direct dielectric barrier discharges (DBDs) (employed in this study) [20,21]. In jets, most of the plasma is generated remotely, and plasma products are often delivered to the biological target via a carrier gas [21]. Ionization waves also produce some plasma at the treatment surface [22]. DBDs generate plasma directly at the surface being treated [20]. Due to the electrode design, DBDs are typically able to a cover a much larger surface area compared to jets [23]. Furthermore, the chemical species deposited on target surfaces, in particular, charged energetic particles and short-lived reactive species, are different between the two systems [20,21,24].

Besides the applied electric field and plasma temperature, a major determinant of the composition of plasma is the gas environment in which the plasma is generated [25]. In the presence of oxygen, reactive oxygen species (ROS) such as atomic oxygen (O), hydroxyl radicals (•OH), superoxide (O_2_•^−^), singlet oxygen (^1^O_2_), and hydrogen peroxide (H_2_O_2_) are produced [26,27,28,29]. When reactive nitrogen species (RNS) are desired for wound healing or other specific applications, nitrogen may be introduced into the discharge environment [30]. Major measured species include atomic nitrogen (N), peroxynitrite (ONOO^−^), and nitric oxide (NO) [31]. Therefore, it is plausible to optimize plasma treatment parameters to produce various chemical effectors for desired biological outcomes.

The highly reactive species are known to influence biological processes and their role in cancer cell killing has been extensively researched [32,33]. In fact, chemotherapies and radiation therapy often rely on the formation of ROS to induce oxidative stress in cancerous cells [34,35]. Photodynamic therapy (PDT) with hypericin (a photosensitizing agent), another treatment for cancers, causes massive production of ROS including ^1^O_2_ in the ER, leading to ICD [36,37]. Typically, DBDs are richer in short-lived reactive species including ^1^O_2_ and •OH, both of which are influencers of biochemical processes [25,38,39]. However, in specific operating modes, certain jets are also able to produce these radicals and other metastable products [40,41]. Consequently, it is important to understand the contribution of short-lived plasma species for ICD induction.

Although mounting evidence in the literature suggests that it is the plasma-generated reactive species that are responsible for provoking cellular responses, electric fields and ultraviolet (UV) light are also produced in plasma, and their interaction with cells and tissue should not be overlooked [24,42]. Plasma-associated pulsed-electric fields may affect cell processes, as electric fields have often been used for electroporation, a process that makes cellular membranes permeable temporarily [43]. Since intracellular changes leading to apoptosis and necrosis can be induced by changing pulsed-electric field characteristics (e.g., pulse duration, electric field intensity, etc.) [43,44,45], we investigated the contribution of electric fields, in our system, on ICD induction.

UV light emitted from plasma may also be crucial for the induction of biological responses, as UV light with wavelengths up to 400 nm are known to induce DNA damage and cell death through multiple pathways [46,47]. Furthermore, Obeid et al. have also reported that ultraviolet C (UV C) is able to induce the emission of ecto-CRT and increase immunogenicity of cancerous cells [48]. Since DBDs have long been used as sources of UV radiation ranging from vacuum UV (110–180 nm) to UV A (320–400 nm) [49], the role of plasma-generated UV for ICD merits investigation.

Altogether, it is clear that plasma is composed of multiple effectors that have been reported to individually induce ICD for cancer immunotherapy. Understanding the role of these plasma components is critical for the development and optimization of plasma technology and deeper mechanistic understanding. Additionally, synergistic effects between plasma components may also provide significant advantages over contemporary therapies.

Numerous studies have reported that plasma changes the oxidative status of cells through the stimulation of intracellular ROS production [24,50,51]. The overall redox status of the cell induces a wide array of cellular responses ranging from proliferation to senescence and death [52,53]. Physiological levels of ROS influence cellular pathways related to growth and proliferation [52]. However, excessive intracellular ROS triggers the oxidative stress pathways that may lead to metabolic alterations and even death [54]. On this spectrum is ER stress, which has been observed in cells undergoing ICD [12,55]. We have reported the upregulation of two ER stress proteins, activating transcription factor 4 (ATF4) and stanniocalcin (STC2), in response to non-thermal plasma exposure of CNE-1, a radiation-resistant nasopharyngeal cancer cell line [56]. These two genes are upstream of ecto-CRT, a critical DAMP signal for ICD [57,58]. Increased secretion of ATP, another DAMP signal and hallmark of ICD, was also observed from cells treated with plasma [56]. Therefore, plasma seems to induce ICD through the canonical cellular oxidative stress pathways.

In this study, we investigated the role of both the plasma-delivered, extracellular ROS and the triggered intracellular ROS for the induction of ICD. Using a nanosecond-pulsed dielectric barrier discharge (nspDBD) plasma, we treated the A549 lung carcinoma cell line and measured one cell-surface DAMP, ecto-CRT, and one released mediator, ATP, as indicators of ICD. To further test the immunogenicity of these cells, we measured the tumor killing capacity of co-cultured macrophages. We assessed the influence of intracellular ROS on ICD by using two chemical ROS attenuators: *N*-acetyl cysteine (NAC) and diphenyleneiodonium (DPI). Since each component of plasma has individually been demonstrated to affect cellular redox, the role of plasma effectors on ICD was evaluated by isolating and removing each sequentially during the treatment of cells.

Our results show that plasma-delivered ROS and charged species increased intracellular ROS and induced DAMP emission. Inhibition of intracellular ROS partially reversed this effect, indicating that plasma triggers cellular oxidative stress pathways. Additionally, ICD induction was accompanied by enhanced anti-tumor activity of macrophages, which was also modulated when the ICD was attenuated. This study describes a new biomedical application of non-thermal plasma which merits further in vivo validation, as plasma could be a safe ICD inducer for cancer immunotherapy.

## 2. Results

### 2.1. NspDBD Plasma Elicits Apoptosis in an Energy-Dependent Manner

**Figure 2:**
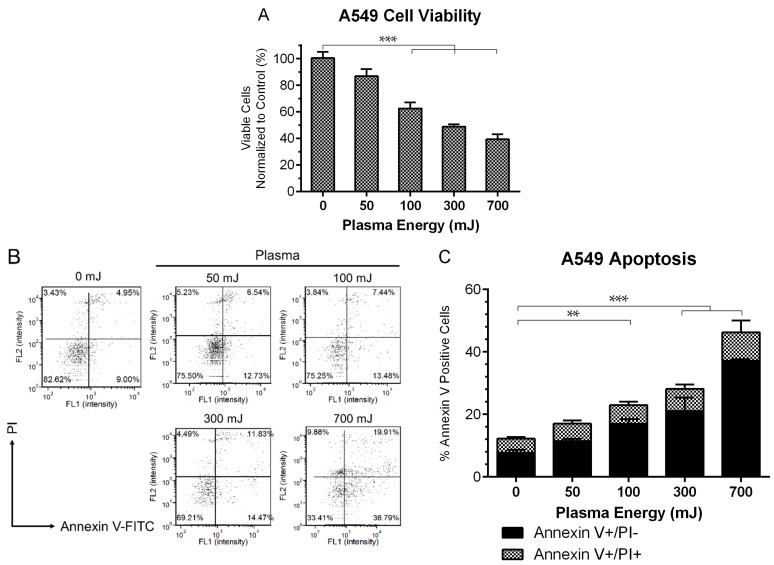
Plasma induced cell death in an energy dependent manner. (**A**) 1 h after plasma exposure, A549 viability decreased as quantified with a propidium iodide (PI) exclusion assay, indicating early damaging effects of plasma (One Way ANOVA, Dunnett’s multiple comparison test). (**B**) Representative dot plots of Annexin V/PI double stained cells 24 h post plasma treatment. Results are summarized in (**C**). Early apoptotic cells (Annexin V+/PI−) were increased significantly compared to untreated controls for all energies above 50 mJ (Two Way ANOVA, Sidak’s multiple comparison test). Data are represented as mean ± SEM. ** *p* < 0.005, *** *p* < 0.001.

Under certain conditions, not fully defined yet, apoptotic cells have been shown to emit DAMPs [16,59]. Therefore, we first examined the effect of different plasma energies on cell viability of the A549 lung carcinoma cell line by exposing them to a range of plasma energies, 50 mJ to 700 mJ as calculated from the treatment time, frequency of pulses, and energy per pulse (described in the Materials and Methods). We have previously shown that with increasing energy, plasma may cause direct cell lysis [42]. Therefore, to assess the early damaging effects of plasma, viable cells were quantified with a Propidium Iodide (PI) exclusion assay, one hour post treatment. Cell viability decreased and the percent of apoptotic cells increased in an energy-dependent manner. At 300 mJ treatment, cell viability was 50% of the untreated cells (Figure 2A). At 24 h post plasma treatment, a statistically significant increase in the number of early apoptotic cells (Annexin V+/PI−) was observed at all energies, 100 mJ and above (Figure 2B,C). Therefore, more cells are likely to express ligands for phagocytic cell receptors for uptake by APCs [60].

### 2.2. NspDBD Plasma Induces Oxidative Stress

The oxidative and reductive (redox) state of the cell is a dynamic balance of oxidants and anti-oxidants [61]. However, when oxidants, either endogenously produced or derived externally, exceed the cell’s anti-oxidant capacity, the result is oxidative stress [53]. Several studies have linked oxidative stress from increased intracellular ROS to the induction of ICD [55,62]. Since most plasma-associated effects are reported to be a result of changing cellular redox [51,63], we explored the role of intracellular ROS in plasma-induced ICD.

**Figure 3:**
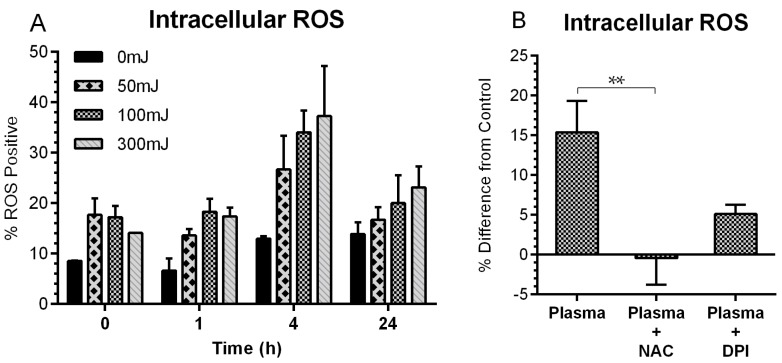
NspDBD-induced oxidative stress is modulated by *N*-acetyl cysteine (NAC) and diphenyleneiodonium (DPI). (**A**) A time course of intracellular reactive oxygen species (ROS) induction following plasma treatment showed the greatest increase of ROS positive cells at 4 h and a subsequent decrease at 24 h. Cell were collected at the indicated times after plasma exposure, stained with 2′,7′-dichlorofluorescein diacetate (DCFDA), and quantified using image cytometry. This experiment was performed once (*n* = 2) to determine the ideal time point to observe changes in intracellular ROS for subsequent experiments. (**B**) Cells pre-incubated in NAC (10 mM) or DPI (5 µM) for 1 h prior to the 300 mJ plasma treatment showed lower levels of intracellular ROS 4 h post plasma (One Way ANOVA, Dunnett’s multiple comparison test). Data are represented as mean ± SEM. ** *p* < 0.005.

A time-course study was carried out to quantify fluctuations in cellular redox following plasma exposure. A549 cells were treated with nspDBD plasma and stained with an intracellular ROS probe, 2′,7′-dichlorofluorescein diacetate (DCFDA), immediately, 1 h, 4 h, and 24-h post exposure. Image cytometry and analysis showed an increase in ROS positive cells with the highest change at 4 h (Figure 3A). By 24 h, intracellular ROS decreased, but did not return to the basal level. To abrogate changes to cellular redox, we incubated cells with 10 mM NAC supplemented media, a scavenger of both plasma delivered and cell generated ROS, 1 h prior to 300 mJ plasma treatment [64,65]. Incubation with 5 µM DPI supplemented media, an inhibitor of nicotinamide adenine dinucleotide phosphate (NADPH) oxidase, 1 h prior to 300 mJ plasma treatment, prevented the generation of intracellular ROS [66]. This energy corresponded to the highest measured ROS. NAC completely reversed the intracellular ROS levels but DPI was only partially effective, indicating that the increase in intracellular ROS after plasma exposure is because of plasma-delivered and plasma-triggered events. Since both agents were efficacious in modulating intracellular ROS (Figure 3B), they were used for subsequent experiments to elucidate the involvement of oxidative stress on plasma-induced ICD.

### 2.3. NspDBD Plasma Elicits Surface Exposure of CRT via Oxidative Stress

**Figure 4:**
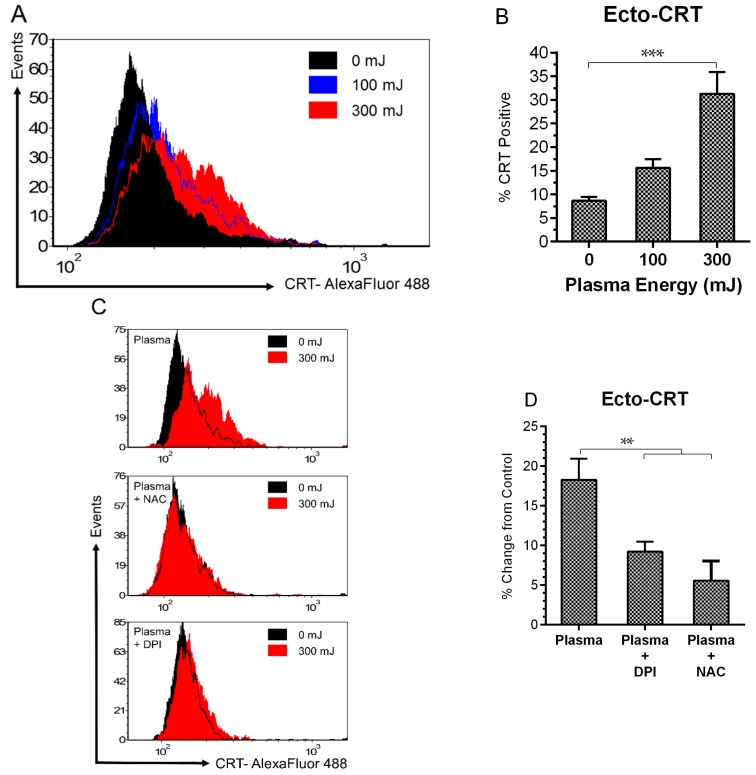
Plasma-elicited emission of surface CRT is associated with oxidative stress. (**A**) Representative histograms of ecto-CRT showed an energy dependent rightward shift in peak fluorescence 24 h following plasma treatment; and (**B**) the percentage of ecto-CRT positive cells increased from 8.6 to 31.2% at 300 mJ (One Way ANOVA, Dunnett’s multiple comparison test); Pre-incubation with NAC and DPI (**C**) inhibited the peak shift and (**D**) reduced the amount of surface-exposed CRT (One Way ANOVA, Dunnett’s multiple comparison test). Data are shown as mean ± SEM. ** *p* < 0.005, *** *p* < 0.001.

Ecto-CRT is a prominent “eat me” DAMP signal that facilitates the engulfment of cells by APCs, such as macrophages and dendritic cells (DCs) [12,16,17,60]. This is followed by their migration to immune organs and processing and presentation of antigens—critical steps for the development of a specific, anti-cancer immune response [7,9,67]. Since both apoptosis and increased intracellular ROS was measured at energies of 100 mJ and 300 mJ, we tested the externalization of CRT at these energies. A statistically significant increase in ecto-CRT was measured at 300 mJ, 24 h post treatment (Figure 4A,B). To determine if ROS is involved in plasma-induced ICD at this energy, we compared the emission of ecto-CRT in the presence and absence of NAC and DPI following plasma exposure. Both NAC and DPI modulated ecto-CRT expression (Figure 4C,D), indicating that plasma-induced CRT emission follows defined oxidative stress pathways.

### 2.4. NspDBD Plasma Elicits Secretion of ATP via Oxidative Stress

**Figure 5:**
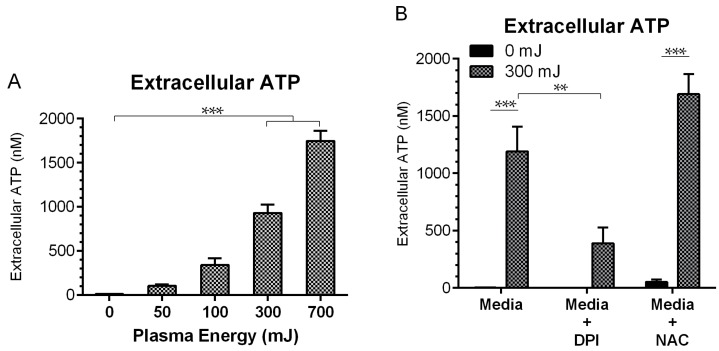
Plasma-elicited ATP secretion is associated with oxidative stress. (**A**) Secreted ATP measured in the supernatant 10 min following plasma treatment increased from 12.3 nM up to 831.2 nM with the 300 mJ treatment (One Way ANOVA, Dunnett’s multiple comparison test). (**B**) DPI was able to temper ATP secretion, while NAC did not (Two Way ANOVA, Sidak’s multiple comparison test). Data are shown as mean ± SEM. ** *p* < 0.005, *** *p* < 0.001.

Extracellular ATP acts as an important “find me” DAMP signal to recruit and activate APCs in the vicinity [13,15,68]. Therefore, secreted ATP was also measured in response to plasma treatment in the presence and absence of NAC and DPI to further establish the integration of oxidative stress pathways in plasma-induced ICD. ATP secreted into the cell culture media was analyzed with a firefly luciferase and luciferin chemiluminescence kit 10 min after the plasma exposure. Extracellular ATP increased significantly, from 12.3 nM in the untreated cells to 831.2 nM following the 300 mJ treatment (Figure 5A). While reduction of the secreted ATP by DPI was statistically significant, NAC had no inhibitory effect on ATP secretion (Figure 5B). This is potentially because ROS-triggered ATP secretion may occur more rapidly than the scavenging effects of NAC.

### 2.5. NspDBD-Elicited ICD Enhances Anti-Tumor Activity of Macrophages

**Figure 6:**
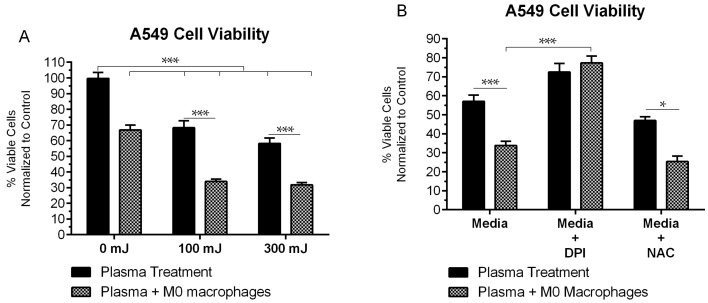
Plasma treatment of tumor cells in immunogenic cell death (ICD)-inducing regimes enhanced anti-tumor activity of macrophages. (**A**) 48 h post plasma treatment and co-culture with M0 macrophages, A549 cells showed reduced viability compared to those treated with plasma and cultured alone. (**B**) Enhanced anti-tumor effects were abrogated when ATP secretion was inhibited with DPI (Two Way ANOVA, Tukey’s multiple comparison test). Data are shown as mean ± SEM. * *p* < 0.05, *** *p* < 0.001.

Cells undergoing ICD become immunogenic and should therefore interact with macrophages to increase their phagocytic activity [69]. Extracellular ATP stimulates DCs and macrophages to release proinflammatory cytokines, such as tumor necrosis factor alpha (TNF-α), interleukin 1 beta (IL-1β), and IL-18, causing tumoricidal activity [70,71,72,73,74]. Therefore, we evaluated the capacity of plasma-stimulated ICD to enhance anti-tumor activity of macrophages. A549 cells were treated at 100 mJ and 300 mJ and cultured with phorbol 12-myristate 13-acetate (PMA)-differentiated THP-1 macrophages (M0 macrophages) in transwell inserts. These inserts separated the two cell types from direct contact but allowed for the exchange of secreted DAMPs from tumor cells and cytokines from macrophages in the media. Quantification of viable A549 cells was performed with the PI exclusion assay 48 h post plasma treatment and co-culture and normalized to untreated A549 cells cultured alone. Since surviving cells continued to proliferate, counts were normalized to the untreated group. In response to plasma alone, 68% and 58% of cells remained viable after the 100 mJ and 300 mJ treatment, respectively. Under co-culture conditions with M0 macrophages, the viability of untreated A549 cells was 65%. This served as the baseline anti-tumor activity of M0 macrophages. Following plasma treatment and co-culture with macrophages, the viability reduced significantly (34% and 32% for the 100 mJ and 300 mJ treatments, respectively), compared to cells treated at the same energy and cultured alone (Figure 6A). In the presence of DPI, where secreted ATP levels are lower (Figure 5B), plasma-treated A549 cells in co-culture showed significantly higher viability compared to those treated and co-cultured in regular media (77% vs. 34%, *p* < 0.001) (Figure 6B). Furthermore, in DPI supplemented media, the difference in viability between plasma-treated A549 cells cultured alone or in the presence of M0 macrophages is insignificant, indicating that the observed cell death is a result of direct plasma treatment. As expected, in the presence of NAC where ATP secretion was not modulated, tumor cell killing by co-cultured macrophages was comparable to that in media (Figure 6B). Based on these results, the observed enhanced anti-tumor activity of macrophages is presumed to be due to the secreted ATP and other DAMPs from cancerous cells undergoing ICD.

### 2.6. NspDBD Plasma-Generated ROS and Charges Are the Major Effectors of ICD

**Figure 7:**
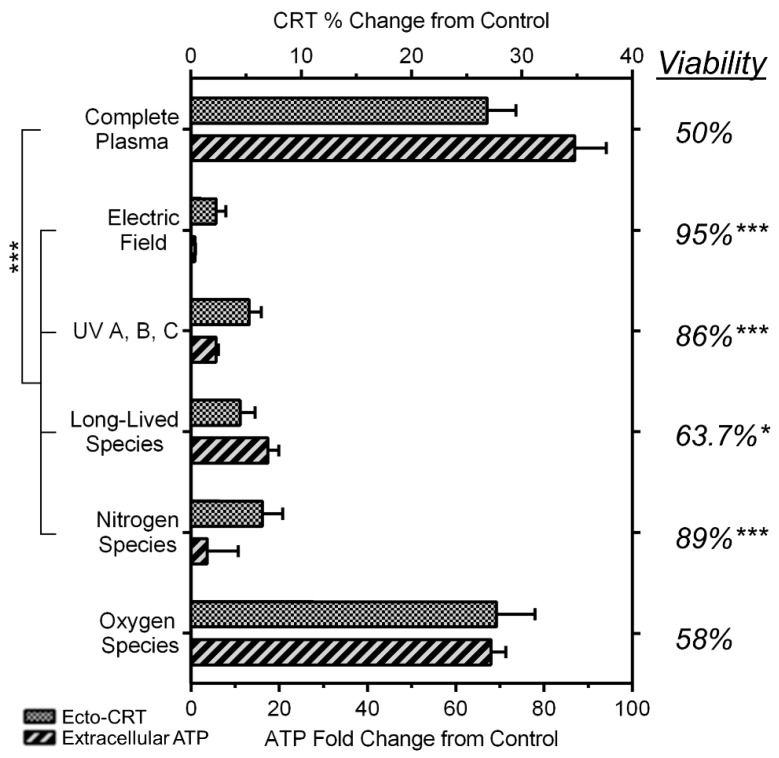
Plasma-generated charges and oxygen species are the major effectors for ICD induction. Components of the complete nspDBD plasma were removed from treatment with physical and gas barriers. Secreted ATP, viability, and ecto-CRT were analyzed at 10 min, 1 h, and 24 h, respectively (One Way ANOVA, Dunnett’s multiple comparison test). Data are shown as mean ± SEM. * *p* < 0.05, *** *p* < 0.001.

When plasma is generated, a complex milieu consisting of electric fields, ultraviolet light (such as UV A, B, C, and vacuum UV), charged species, and neutral gas species is produced [24,38,75,76]. To identify which of these is the major contributor of plasma-induced ICD, we dissected the plasma using barriers and treatment conditions engineered to remove components from the treatment (Materials and Methods). Parameters that generated a plasma discharge at 300 mJ were used for all treatment conditions (29 kV, 30 Hz, 10 s treatment time, and 1 mm application distance) and ecto-CRT and secreted ATP were measured as indicators of ICD. Results are expressed as percent (CRT) or fold (ATP) change with respect to mock-treated cells, which served as our negative control. Cells exposed to complete plasma served as the positive controls and correspond to 50% cell death, 27% increase in ecto-CRT positive cells, and an 87-fold increase in secreted ATP (Figure 7).

**Figure A1:**
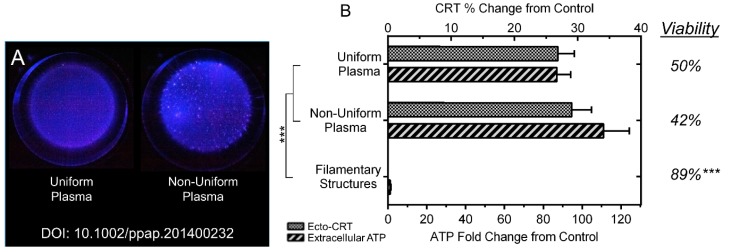
(**A**) Filamentary structures of non-uniform plasma can be observed as bright pixels in the presented photographs of plasma discharges (taken from our previous publication; methods and photograph parameters are detailed there [42]); (**B**) Non-uniform plasma treatment was performed in air (labeled “Non-Uniform Plasma”) and nitrogen (labeled “Filamentary Structures”) and compared to the uniform treatment to compare the two regimes (One Way ANOVA, Tukey’s multiple comparison test). Data are shown as mean ± SEM. *** *p* < 0.001.

To examine the contribution of global electric fields and UV light, a liquid barrier and a quartz barrier were employed, respectively. When the electrode is submerged in a liquid, the applied electric field is less than the dielectric strength of the medium. Therefore, while electric fields are present, plasma is not generated (Appendix A). Quartz prevents penetration of all plasma components except UV A, B, and C. Our results show that in both cases, cell viability and DAMP emission remained similar to the untreated controls. The lack of effect of local electric fields within the plasma was also observed when plasma was operated in different modes of DBD (Figure A1). This implies that plasma-associated electric fields and UVs have negligible effects on ICD induction.

When a mesh barrier is used, plasma is generated between the electrode and the mesh and only the long-lived species may be delivered to the cells. In general, secondary plasma generation may also occur between the mesh and the cells but it was not visibly observed in this case. When we used the mesh barrier, cell viability recovered marginally (64% viable cells from 50%) as compared to those treated with all plasma components. However, DAMP emission decreased significantly. CRT decreased from 27% to 4% and ATP went down from 87-fold to 17-fold. This strongly suggests that while long-lived species (e.g., H_2_O_2_, NO, O_3_, etc.) are crucial factors causing cell death, two components vital to direct cells toward the ICD pathway are charged and short-lived reactive species (e.g., ^1^O_2_, •OH, O_2_•^−^, etc.).

Both RNS and ROS affect biological processes and cellular functions [77]. Since our plasma treatment is performed in air composed mostly of nitrogen and oxygen, we studied the individual effects of plasma-generated nitrogen and oxygen species. Cells were treated in a pure nitrogen or pure oxygen environment following evacuation of air from the well. Because species produced by plasma are highly sensitive to the gas in their environment [49,78], in each case the plasma would produce either nitrogen or oxygen species only, respectively. Our results showed that cell viability remained high when plasma was generated in nitrogen and no change in DAMP signal expression was observed, signifying no role of nitrogen species in this process. However, when plasma was generated in oxygen, the effects on cells were similar to those in air. The viability increased marginally from 50% to 58% under the oxygen environment and ecto-CRT remained similar (27% and 28%). The change in ATP emission was more noticeable, though not statistically significant (87-fold and 68-fold, complete plasma vs. oxygen species, respectively). Nitrogen plasma retains certain charged species, e.g., electrons, electronically excited N_2_, N^+^ ions, etc. [24]. The insignificant changes observed above, suggest that charges by themselves are not sufficient to elicit ICD but require the presence of oxygen species. Taken together, the major effectors of plasma-induced ICD are identified to be: (1) plasma-generated ROS and (2) charged species.

## 3. Discussion

**Figure 8:**
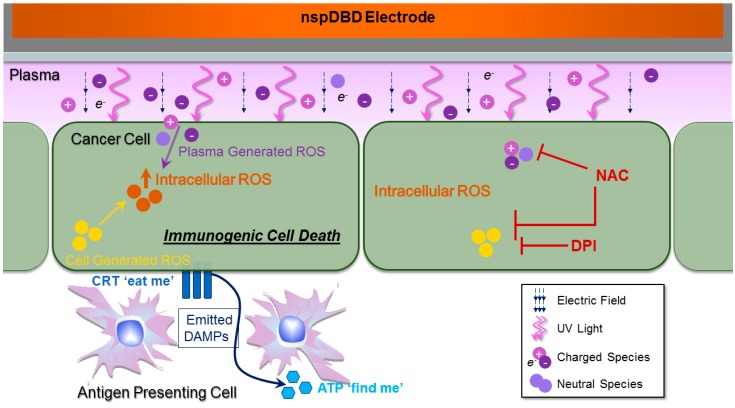
Plasma and cancer cell interaction for the induction of immunogenic cell death. When plasma comes in contact with a biological target, four major effectors are delivered: electric fields, UV light, and charged and neutral species. Plasma-generated ROS and charged species are the major contributors for eliciting ICD. Following plasma exposure, damage associated molecular patterns (DAMP) signals (ecto-CRT and secreted ATP) are emitted. Extracellular ATP acts as a ‘find me’ DAMP signal to recruit immune cells to the vicinity and activate them [68]. Surface-exposed CRT serves as an “eat me” signal and mediates the engulfment of tumor cells by antigen presenting cells (APC) [16]. Plasma-induced DAMP emission is associated with increased intracellular ROS and may result from the diffusion of plasma-generated species, or from the stimulation of the cell’s own ROS-generating mechanisms. DPI was used to inhibit cellular ROS production, while NAC was used to scavenge both extracellularly diffused and intracellular generated ROS. Both ROS attenuators abrogated intracellular ROS and surface CRT expression. (Abbreviations: ATP, adenosine triphosphate; CRT, calreticulin; DPI, diphenyleneiodonium; NAC, *N*-acetyl-l-cysteine; nspDBD, nanosecond-pulsed dielectric barrier discharge; ROS, reactive oxygen species.) T-bars indicate site of ROS inhibition/scavenging.

In this study, we investigated the interaction of two intricate systems, plasma and cancer cells, for the initiation of ICD through oxidative stress (Figure 8). The induction of immunogenic death in cancer cells has great potential for amplifying anti-cancer immune processes and improving clinical efficacy with the added benefit of fewer adverse effects [1]. ICD involves changes to the surface composition of the cell and the secretion of soluble factors [5]. The use of non-thermal plasma is a novel approach to elicit the emission of danger signals, known as DAMPs, characteristic of ICD [56]. We have tested DAMP emission in other cell lines including, CNE-1 (nasopharyngeal carcinoma), CT26 (colorectal cancer), and more recently Panc02 (pancreatic adenocarcinoma), but in depth analysis is required to validate that the effects are plasma-induced. The contribution of each plasma component to ICD induction was investigated and the cellular responses to plasma manipulation were measured. This lays the foundation for future development of plasma technology for ICD-mediated cancer immunotherapy.

Plasma is a complex milieu of electric fields, ultraviolet radiation, and charged and neutral species. For the induction of ICD, we showed that the key plasma effectors are short-lived, charged, and neutral ROS (Figure 7). In our system, applied electric fields do not contribute to plasma-induced ICD; this can be attributed to the pulse parameters of our nspDBD system. Although pulsed-electric fields have been shown to cause cell death, it is highly dependent on pulse amplitude and duration [45,79,80,81]. Beebe et al. have shown that applied electric fields at 300 kV/cm with pulse widths between 10–60 ns did not affect cell membrane integrity [44]. The pulse width of our plasma regime (20 ns at 300 kV/cm) are within this range and are therefore insufficient to alter cell functions by themselves. However, plasma with different voltage pulse characteristics will have different applied electric fields resulting in other biological outcomes (e.g., reversible electropermeabilization, cell death, and inactivation of bacteria) [81,82,83]. The role of plasma-generated UV A, B, and C also appear to be insignificant for the induction of ICD. Obeid et al. reported that UV C is a robust ICD inducer: cancer cells irradiated at 100 J/cm^2^ expressed ecto-CRT as early as 1 h post treatment and persisted for 24 h [48]. In our plasma system, UV power is approximately 15 µW/cm^2^ as measured with a vacuum photodiode and radiometer (InternationalLight Technologies, Peabody, MA, USA). Over the 10 s treatment time, cells experienced approximately 150 µJ/cm^2^ of UV radiation from plasma, which is orders of magnitude less than the reported amount required for UV C-induced ICD. Therefore, we conclude that in our nspDBD regime, plasma-induced ICD is a result of plasma-produced chemical species. Comparing cell response to plasma discharges in nitrogen, oxygen, and air, we conclude that the charges and short-lived oxygen species are required for the induction of ICD with nspDBD plasma. Some of these species include ^1^O_2_, •OH, and O_2_•^−^ as identified through computational modeling, optical emission spectroscopy of plasma, and liquid analysis with plasma-treated solutions [24,31,39,84]. Delineation of the exact species responsible is required for understanding the underlying mechanisms of plasma and cell interactions and for additional optimization and development of plasma systems for medical applications. Furthermore, the effect of RNS (e.g., NO, ONOO^−^, etc.) which requires both oxygen and nitrogen should also be studied, as their contribution cannot be elucidated through the physical barriers or gas conditions used here. The use of specific scavengers may be employed for a detailed examination of those individual species.

The oxidative stress from plasma treatment may result from the diffusion of plasma-generated species, or from stimulation of the cell’s own ROS generating mechanisms. In order to analyze the biochemical response of cancer cells to plasma and its correlation to DAMP emission, we used two chemical agents, DPI and NAC to abrogate intracellular ROS. Since plasma treatments were done in the absence of liquid, cells were incubated in the presence of DPI or NAC, both before and after treatment. This was done to avoid any modifications by plasma to the two inhibitors. DPI is an inhibitor of NADPH oxidase, an enzyme whose primary function is to generate intracellular ROS for cellular metabolism [66,85]. The pre-treatment of cells with DPI prevented the rise of intracellular ROS associated with plasma exposure (Figure 3B), and also reduced the externalization of CRT and secretion of ATP (Figure 4C,D and Figure 5B). However, neither intracellular ROS nor measured DAMP signals returned to baseline with the use of DPI. This may be explained by the fact that in addition to NADPH oxidase, mitochondria are an important source of intracellular ROS. They are also a key effector of apoptosis and the site of cellular ATP synthesis [86,87]. Plasma increases ROS production by mitochondria but DPI is not known to inhibit mitochondrial enzymes [50]. Some of the measured intracellular ROS may also be from the diffusion of plasma-generated species. Furthermore, exogenously generated ROS may be able to elicit ICD to some degree, though the effects are more pronounced when the cell’s metabolic ROS mechanisms have been stimulated.

NAC is a precursor of glutathione that decreases oxidative stress by directly neutralizing ROS [64,65]. It scavenges both plasma-produced and cell-generated intracellular ROS, whether from NAPDH oxidase or the mitochondria [50]. We observed that intracellular ROS levels of plasma-treated cells were similar to those of untreated cells, when cells were pre-incubated with NAC (Figure 3B). This further supports our assumption that plasma can modulate cellular redox by both exogenously delivered and endogenously stimulated ROS. NAC was also able to abrogate the emission of ecto-CRT (Figure 4C,D), which is translocated to the cell surface when cells undergo ER stress with concomitant intracellular ROS generation [4,5]. Previously, we have shown that non-thermal plasma operated in the ICD-inducing regime, can upregulate ER stress genes associated with CRT translocation [56]. Our results here with the use of scavengers, provide strong evidence that plasma-induced emission of ecto-CRT requires the participation of oxidative and ER stress pathways.

NAC did not reduce the plasma-induced secretion of ATP while DPI did (Figure 5B). This could be attributed to the different mechanisms by which NAC and DPI attenuate intracellular ROS and the temporal progression of ATP secretion pathways. Sun et al. studied the effects of *Clostridium difficile* toxin B for the induction of intracellular ROS and ICD and reported similar observations [62].

Altogether, we observed that treatment conditions involving physical barriers, controlled atmosphere, and chemical attenuators, did not revert emission of DAMP signals back to physiological levels. This suggests that plasma effects are not a result of any individual component, but rather, cancer cell interactions with plasma must involve multiple, complex pathways in addition to the modification of ROS. Thus, a combination of plasma effectors is required for ICD induction in cancer cells.

We also demonstrated that emitted ATP from plasma-treated cells undergoing immunogenic death was immuno-stimulatory through our co-culture experiments with macrophages. Macrophages were inhibitory to tumor cells in our system (Figure 6A). However, ICD induction in tumor cells magnified this anti-tumor effect of macrophages. Plasma-treated A549 cells secrete ATP, but other DAMPs secreted in response to oxidative stress include HMGB1, HSP70, and HSP90 [88]. We measured fluctuations in ATP levels as a representative secreted DAMP. Extracellular ATP stimulates macrophage secretion of TNF-α, IL-1β, and other cytokines that are cytotoxic to cancerous cells [15,70,73,89]. As a result, plasma-treated A549 cell viability was decreased when cultured in the presence of macrophages compared to that of cells cultured alone (Figure 6A). This effect was reversed when ATP was inhibited by DPI (Figure 6B). Our results provide evidence that plasma can be a potential immuno-stimulator for cancer therapy. Bundscherer et al. have previously shown the effect of plasma on mitogen-activated protein kinase (MAPK) signaling pathways that are involved in all aspects of immune response [90]. A recent study using an animal model of melanoma also highlights plasma’s ability to modulate immune function for the control of tumors [91].

Plasma for cancer treatment has focused mainly on its ablative properties, utilizing energies where damage to tumor cells exceeds harmful effects to surrounding normal tissue [92,93,94,95,96,97]. While the results seem promising, in animal studies, tumors recurred when treatment was discontinued and there was visible and histological damage in the treatment area [92,98]. In this study, we used stress-inducing regimes of plasma on cells in contrast to those previously reported [56,99]. We show that plasma can act as an adjuvant by eliciting immunogenic cancer cell death for the engagement of the patient’s immune system to eradicate cancer. We have also previously demonstrated that plasma can directly augment macrophage function. In vitro, we demonstrated that the migratory activity and secretion of anti-tumor cytokines from macrophages were enhanced following plasma stimulation [56,100,101,102]. In vivo, we showed that plasma treatment enhanced the differentiation of hemocytes in the lymph organs of *Drosophila melanogaster* larvae [103]. Augmented recruitment of myeloid APCs one week after a single treatment was also demonstrated by our group in the treated skin of live mini-pigs [104]. Therefore, plasma not only stimulates APCs directly, but also indirectly through the induction of ICD on tumors. We suggest that plasma may synergistically boost anti-cancer immune responses through more efficient uptake of cancerous cells, leading to exposure of neoantigens from the tumors and allowing for the development of a robust new T-cell response.

These initial in vitro results have been expanded and validated in an in vivo study of murine colorectal cancer using the CT26 cell line (manuscript in preparation). Included is a vaccination assay, the “gold-standard” test that shows that plasma is a bone fide ICD-inducer [2,11,105]. Our results support the feasibility and clinical potential of plasma-induced ICD for cancer immunotherapy.

## 4. Materials and Methods

### 4.1. Cell Culture and Plating

Human lung carcinoma cells (A549, ATCC:CCL-185) were a gift from Bela Peethambaran (University of the Sciences, Philadelphia, PA, USA) and were cultured in RPMI 1640 with 10% fetal bovine serum and 1% penicillin/streptomycin (Corning Life Sciences, Corning, NY, USA). The human monocyte cell line (THP-1, ATCC:TIB-202) was a gift from Kara Spiller (Drexel University, Philadelphia, PA, USA). THP-1 cells were cultured in complete media: RPMI 1640 with 10% heat inactivated fetal bovine serum and 1% penicillin/streptomycin (Corning Life Sciences). All cells were grown at 37 °C with 5% CO_2_ in a humidified atmosphere (Panasonic, MCO-19AICUVH-PA, Wood Dale, IL, USA).

A549 cells were plated into 24-well plates at 3.0 × 10^5^ cells/mL (0.5 mL/well) or into 6-well plates at 4.0 × 10^5^ cells/mL (2 mL/well) one day prior to plasma treatment. Before plasma treatment, the media was removed and the cells were washed twice with phosphate buffered saline (PBS). PBS from the second wash was removed right before plasma treatment and 500 µL or 2 mL of complete cell culture media was added immediately after cells were exposed to plasma in 24- or 6-well plates, respectively.

For the co-culture experiments, THP-1 monocytes were seeded into transwell inserts (0.4 µm pore size) (Corning Life Sciences) at 1.6 × 10^5^ cells/mL and differentiated into M0 macrophages with 0.5 µL of 100 µL/mL phorbol 12-myristate 13-acetate (Sigma-Aldrich, St. Louis, MO, USA). Inserts were placed into a 24-well plate with 600 µL of THP-1 complete media, and cultured separately in a humidified environment at 37 °C with 5% CO_2_ overnight. Following incubation, cells were washed with PBS before co-culture. Immediately following plasma treatment of the A549 cells, 750 µL of complete media was added to each well and M0 macrophages in transwell inserts were transferred to each well. An additional 750 µL of THP-1 complete media was added to the inserts. Media was changed at 24 h with fresh complete media, and cells were cultured for an additional 24 h before viability analysis.

### 4.2. NspDBD Plasma Treatment Parameters

**Figure 9:**
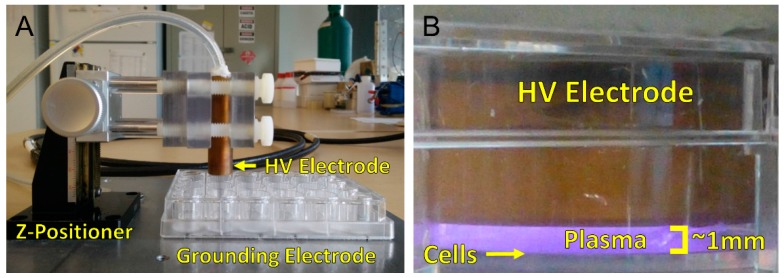
NspDBD plasma treatment system. (**A**) Plasma is generated between the high voltage (HV) electrode and the cells in a well. A z-positioner was used to standardize the plasma application distance; (**B**) A 1 mm gap was maintained between the electrode and the cells on the bottom of the plate.

All plasma treatments of cells were performed in the absence of liquid. Media was removed and cells were washed with PBS immediately prior to plasma exposure and media was immediately added back. The cells were treated in a humidity controlled environment (~60% relative humidity). Non-thermal plasma was produced by applying a high voltage pulse to a DBD electrode 1 mm above the cells in the wells. The DBD electrode used for all experiments without physical barriers, was 1.3 cm in diameter and fit into the wells on a 24-well plate. The wells rested on a grounding plate that acted as our second electrode, and plasma was generated in the gap between the electrode and the plate, in direct contact with the cells (Figure 9). When the mesh and quartz barriers were used, more space between the electrode and the walls of the well was required. For those studies, cells were treated in 6-well plates (3.5 cm diameter wells) with a 2.5 cm diameter electrode, to accommodate the space required for the barrier. Plasma energies were measured with the 6-well plate and treatment parameters were re-calculated to account for this changed configuration. In this set up, the plasma was generated between the electrode and the mesh, right above the cells.

The characteristics of our plasma pulse and the methods of measuring energy of a single discharge have been defined in our previous work [42]. In this study, we used a nanosecond pulser (FPB-20-05NM, FID GmbH, Burbach, Germany) that generated 29 kV pulses with 2 ns rise times and 20 ns total pulse duration. The duration of all our treatments was fixed at 10 s, and the pulse frequency was controlled with an external function generator (TTi, TG5011 LXT, Philadelphia, PA, USA). Total plasma energy delivered to the cells was calculated from the treatment time, frequency of pulses, and energy per pulse. Since the energy per pulse is dependent on the discharge surface (polystyrene plate vs. grounded copper mesh), for some experiments the frequency was recalculated to achieve comparable plasma treatment energies. Table 1 depicts the operating parameters for our plasma treatments.

### 4.3. Removal of Plasma Effectors to Determine the Major Contributors of Plasma-Induced ICD

We operated our nspDBD at 300 mJ to determine the relative contribution of plasma effectors on ICD. The barriers and conditions designed to remove specific plasma effectors delivered to cells during the treatment have been detailed in our previous study and are summarized in Table 2 [42]. Briefly, the direct nspDBD in air contained all plasma effectors (complete plasma) and was used as our positive control for all experiments. To determine the contribution of the electric fields, we did not remove PBS prior to treatment; the electrode was dipped into PBS, positioned 1 mm above the cells, and operated at a 30 Hz pulse frequency, corresponding to 300 mJ of plasma treatment. Therefore, with the same applied voltage, the plasma was eliminated while the applied electric field remained [106]. The contribution of plasma-generated UV A, B, and C was determined by containing the plasma between a grounded copper mesh and quartz barrier. Since quartz is only transparent to wavelengths greater than 200 nm (Technical Glass Products, Inc., Fused Silica, Painesville Twp., OH, USA), this barrier allows only UV light to be delivered to the cells. The grounded mesh barrier, without the quartz, allowed for the long-lived neutral species to be delivered to cells during treatment, while removing charged species and short-lived species [25]. Since the energy per pulse of plasma generated on the copper mesh was 1.9 mJ/pulse, the frequency was adjusted to 15 Hz to keep plasma treatment energy the same (300 mJ) for those two conditions (Table 1). Most DBD treatments are done in atmospheric air, composed mainly of oxygen and nitrogen, so we examined the effect of oxygen species by inserting the electrode into the well and sealing it. We flowed 99.999% pure oxygen (Airgas, Bellmawr, NJ, USA) into the well through a 22-gauge inlet needle at a rate of 2 standard cubic feet per minute (SCFM) for 5 s; an outlet needle was placed on the other side to allow the existing air to be displaced. Plasma discharged in this oxygen environment eliminated nitrogen and other non-oxygen chemical species. To determine the effect of nitrogen species, 99.999% pure nitrogen (Airgas) was passed into the well similar to oxygen in the previous setup, thus removing plasma-generated oxygen species. Plasma exposure under these two conditions helped elucidate the contribution of plasma-generated oxygen and nitrogen species for ICD induction.

### 4.4. UV Power Measurements

UV light emitted from our plasma discharge was measured with a vacuum photodiode (InternationalLight Technologies, SED220) and radiometer (InternationalLight Technologies, IL1700). The photodiode has a fused silica window and an active area of 50 mm^2^. The detector was placed parallel to the grounded copper mesh barrier with the electrode discharging 1 mm above it at the 300 mJ treatment parameters. The radiometer was operated in the DC signal mode with 0.5 s sampling times and zeroed to ambient room conditions before every run. Ten readings were taken and the data were reported as µW/cm^2^. The addition of quartz to the mesh barrier did not attenuate UV power density.

### 4.5. Pre-Treatment with NAC and DPI to Attenuate Intracellular ROS

Medium supplemented with 10 mM NAC (Sigma-Aldrich) or 5 µM DPI (Sigma-Aldrich) was used to culture the cells 1 h before plasma exposure. A dose response was performed for both ROS attenuators to select the concentration required to modulate the oxidative stress based on previous studies [62,107]. Cells were then washed twice with PBS (with calcium and magnesium) supplemented with NAC and DPI at the same concentrations. Immediately after plasma exposure, fresh media with the ROS attenuators was added back into the well for up to 24 h. For co-culture experiments only, the media was changed at 24 h, and cells were cultured for an additional 24 h in the absence of ROS attenuators.

### 4.6. Quantification of Cell Viability

A Propidium Iodide (PI) (Thermo Fisher Scientific Inc., Waltham, MA, USA) exclusion assay was used to assess cell viability. PI is a DNA-binding fluorescent agent that penetrates damaged cell membranes. Cells were trypsinized 1 h post plasma treatment and stained with 100 µL/mL of PI (Invitrogen, Waltham, MA, USA) to assess the early damaging effects of plasma. Live cells were quantified with an image cytometer (Nexcelom CBA Vision, Nexcelom Bioscience, Lawrence, MA, USA) and additional analysis, including size gating, was performed using the FCS flow cytometry software (FCS 4.0, DeNovo Software, Glendale, CA, USA). Viability was determined by normalizing live cell counts of plasma treated samples with mean live cell counts of untreated controls. The data represent viable cells as a percentage of the controls.

### 4.7. Quantification of Apoptotic and Necrotic Cells

Apoptosis at 24 h post treatment was determined by measuring the surface exposure of phosphatidylserine (PS) with an Annexin V-PI apoptosis assay. Cells were collected, washed with PBS, pelleted, and resuspended in 100 µL of 1× Annexin V binding buffer (Thermo Fisher Scientific Inc.). Then 5 µL of Annexin V, Alexa Fluor 488 conjugate (Thermo Fisher Scientific Inc.), and 2 µL of PI (1 mg/mL) were added to the cell suspension and incubated at room temperature in the dark for 15 min. Following incubation, the cells were washed twice with 1× Annexin V binding buffer and the image cytometer was used to measure fluorescence. Annexin V+/PI− cells indicate early apoptosis, Annexin V+/PI+ cells indicate late apoptosis, and Annexin V−/PI+ cells indicate necrosis. Dot plots were made with the FCS software version 4 and the percentage of Annexin V positive and PI positive cells were graphed.

### 4.8. Quantification of Extracellular ATP

Ten min following the plasma treatment, 50 µL of media was collected and extracellular ATP was quantified using an adenosine 5′-triphosphate chemiluminescent somatic cell assay kit (Sigma-Aldrich). All reagents were prepared following the manufacturer instructions. ATP assay mix stock solution was diluted at a ratio of 1:25 with dilution buffer to prepare the working solution. One hundred µL of working solution was added to 12 mm × 55 mm test tubes (Hach, Springfield, MO, USA) and allowed to stand at room temperature for 3 min before use. Fifty µL of the collected cell supernatant was diluted with 100 µL of ultrapure water (Sigma-Aldrich) and 50 µL of the diluted supernatant was transferred into the 12 mm × 55 mm test tubes containing the ATP assay mix working solution. The assay mix, containing firefly luciferase and luciferin, catalyzes the reaction between ATP and luciferin to form adenyl-luciferin. Adenly-luciferin goes on to react with oxygen to produce oxyluciferin, adenosine monophosphate, carbon dioxide, and light, which is measured in a luminometer (Photon-Master, LuminUltra, Springfield, MO, USA). The luminometer was calibrated with the provided UltraClear calibration solution and the measured relative light units (RLU) are converted into pgATP/mL. The RLU from the samples were measured with the Photon-Master immediately following mixing with the ATP assay mix in the test tubes and the extracellular ATP concentration was determined. Data were represented as either the ATP concentration (nM) or the fold change of untreated controls.

### 4.9. Fluorescence Detection of Ecto-CRT

24 h after plasma treatment, A549 cells were collected and washed with blocking buffer (2% FBS in PBS) and incubated with rabbit anti-human calreticulin antibody (Thermo Fisher Scientific) in blocking buffer (1:200 dilution) at room temperature, in the dark, for 30 min. The cells were then washed twice with blocking buffer and incubated with Alexa Fluor 488 conjugated goat anti-rabbit IgG (Thermo Fisher Scientific.) secondary antibody (1:500 dilution in blocking buffer) in the dark, at room temperature, for 40 min. At the end of the incubation, the cells were washed twice and analyzed by image cytometry and FCS software version 4. Representative histograms were plotted and normalized to the number of cells detected. Data are represented as the percentage of cells positive for surface CRT staining or the difference in percent from the untreated cells.

### 4.10. Statistical Analysis

Each experiment was performed with a minimum of three replicates and repeated at least twice unless otherwise stated. Data are presented as mean ± SEM. Statistical analysis was performed in GraphPad Prism 6 (GraphPad Software, La Jolla, CA, USA) using one-way ANOVA with post hoc Dunnett’s or Tukey’s multiple comparisons test. A two-way ANOVA test was also used with post hoc Sidak’s or Tukey’s multiple comparisons test. *p* < 0.05 Was considered significant. Only statistically significant comparisons are indicated in the figures.

## 5. Conclusions

Immunotherapy is becoming the treatment of choice for all cancers where this option is available but is fraught with serious adverse effects, even death. ICD-mediated pathways offer a safer option whereby cancer cells are forced into an atypical cell death pathway that allows APCs to easily recognize and digest them, making them more immunogenic. We used non-thermal plasma, an efficient and controllable ROS delivery system, to induce ICD in A549 lung cancer cells. We show that these cells emit two characteristic molecules, ATP and ecto-CRT for the engagement of APCs with subsequent killing of tumor cells by APC-released molecules. This is the first report showing a directed immunomodulation via non-thermal plasma for treatment of cancer.

## Figures and Tables

**Table 1:** Nanosecond Pulsed Dielectric Barrier Discharge Plasma Parameters.

Parameter	Value
Excitation	Nanosecond-pulsed
Voltage	29 kV
Rise Time	2 ns
Pulse Width	20 ns
Treatment Time	10 s
**Plasma Discharge in 24-Well Plate (Energy per Pulse: 0.9 mJ/pulse)**	
Gap Distance	1 mm
Frequency	5, 15, 30, 75 Hz
Total Plasma Energies	50, 100, 300, 700 mJ
**Plasma Discharge on Copper Mesh Barrier (Energy per Pulse: 1.9 mJ/pulse)**	
Gap Distance	1 mm
Frequency	15 Hz
Total Plasma Energy	300 mJ

**Table 2:** Summary of treatment conditions and delivered plasma effectors.

Treatment Condition			Effectors Delivered	Effectors Removed
1.	NspDBD in Air	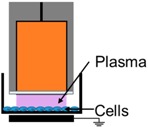	E-Field, UV, Charges, Neutrals	-
2.	Electrode Dipped in Media	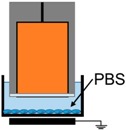	E-Field	UV, Charges, Neutrals
3.	NspDBD w/Quartz Barrier	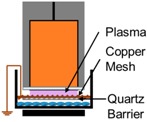	UV	E-Field, Charges, Neutrals
4.	NspDBD w/Mesh Barrier	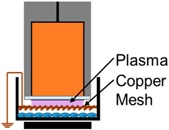	Long-Lived Neutrals, UV	Global E-Field, Charges, Short-Lived Neutrals
5.	NspDBD in Oxygen	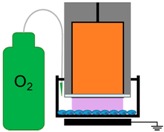	ROS, Charges, E-Field, UV	Other Neutral Species
6.	NspDBD in Nitrogen	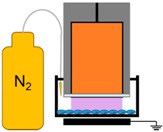	Nitrogen Species, Charges, E-Field, UV	Other Neutral Species

## Abbreviations

ICD	Immunogenic cell death
APC	Antigen presenting cell
DAMP	Damage associated molecular pattern
ER	Endoplasmic reticulum
ROS	Reactive oxygen specie
CRT	Calreticulin
Ecto-CRT	Surface-exposed calreticulin
ATP	Adenosine triphosphate
HMGB1	High mobility group protein B1
HSP90	Heat shock protein 90
HSP70	Heat shock protein 70
HV	High voltage
RNS	Reactive nitrogen specie
DBD	Dielectric barrier discharge
ATF4	Activating transcription factor 4
STC2	Stanniocalcin
NspDBD	Nanosecond-pulsed DBD
NAC	*N*-acetyl cysteine
DPI	Diphenyleneiodonium
PI	Propidium iodide
DCFDA	2′,7′-dichlorofluorescein diacetate
NADPH	Nicotinamide adenine dinucleotide phosphate
DC	Dendritic cell
TNF-α	Tumor necrosis factor α
IL-1β	Interleukin 1β
IL-18	Interleukin 18
PMA	Phorbol 12-myristate 13-acetate

## Appendix A

DBD plasma exposure to cells in vitro often involves the treatment of relatively smooth surfaces such as plates or petri dishes. However, when surfaces become topographically uneven, plasma can concentrate on “ridges” (where the gap distance is minimal), resulting in a non-uniform discharge [108]. This becomes particularly problematic in subnanosecond-pulsed DBDs as plasma in this non-uniform regime produce filamentary structures that may cause highly localized heating and damage [109]. However, by applying high electric fields (~30 kV/cm) over nanosecond rise times, a more uniform discharge is achieved, even over uneven surfaces, thus reducing injurious effects of filaments [108,110]. Using fast intensified charge-coupled device (ICCD) imaging, Liu et al. observed the transition from non-uniform to uniform discharge and quantified uniformity with a chi-square test [110]. Furthermore, using spectroscopic measurements of electric field in the discharge, they report that filamentary structures create strong electric fields locally within plasma, resulting in distinctly higher electric fields in the non-uniform regime compared to that of the uniform regime [110]. This has been reported both experimentally and in simulations, for plasma jets and DBDs [111,112,113]. Due to the close relationship between electric fields and plasma chemistry, changing the mode of plasma could also influence biological effects [39,113]. Therefore, the uniformity of plasma for induction of ICD requires investigation.

To determine the effect of plasma uniformity on the induction of ICD, we compared the outcomes of the nspDBD system operated in the uniform and non-uniform regime (Figure A1A). NspDBD plasma transitions into a filamentary, non-uniform regime when the applied electric field in the discharge gap is reduced [110]. In our system, this occurs when the gap distance is increased from 1 mm to 2 mm [110]. Since the energy per pulse of plasma in the non-uniform regime was 0.4 mJ/pulse, as measured in our previous publications, frequency was adjusted to 75 Hz to ensure that the total plasma energy delivered to cells during treatment was kept at 300 mJ [42]. Plasma, in both regimes, was discharged in air and nitrogen. When plasma is discharged in nitrogen gas, the influence of local electric fields from filaments can be delineated [42]. We observed no significant difference between the two regimes of plasma in air on cell death and DAMP response (Figure A1B). However, non-uniform discharge in nitrogen did not affect viability or cause ICD, meaning that higher electric fields from filamentary structures in the non-uniform regime are inconsequential.

For in vivo treatment with DBD plasma, surfaces are never completely smooth so transition to the non-uniform regime may be inevitable. Our results indicate that the induction of ICD may be achieved at multiple DBD plasma regimes and is crucial for the translation of plasma technology for clinical applications.
